# Metabolomics Reveal the Regulatory Effect of Polysaccharides from Fermented Barley Bran Extract on Lipid Accumulation in HepG2 Cells

**DOI:** 10.3390/metabo13020223

**Published:** 2023-02-03

**Authors:** Yan-Sheng Zhao, Xin-Meng Tong, Xue-Mei Wu, Juan Bai, Song-Tao Fan, Ying Zhu, Jia-Yan Zhang, Xiang Xiao

**Affiliations:** School of Food and Biological Engineering, Jiangsu University, Zhenjiang 212013, China

**Keywords:** metabolomics, polysaccharides, barley bran, lipid accumulation, *Lactiplantibacillus plantarum*

## Abstract

Barley bran has potential bioactivities due to its high content of polyphenols and dietary fiber, etc. Fermentation has been considered as an effective way to promote the functional activity of food raw materials. In this study, polysaccharides from barley bran extract fermented by *Lactiplantibacillus plantarum* dy-1 (FBBE-PS) were analyzed, and its effects on lipid accumulation and oxidative stress in high-fat HepG2 cells induced by sodium oleate were evaluated. The results showed that the molecular weight decreased and monosaccharide composition of polysaccharides changed significantly after fermentation. In addition, 50 μg/mL FBBE-PS could reduce the triglyceride (TG) content and reaction oxygen species (ROS) level in high-fat HepG2 cells by 21.62% and 30.01%, respectively, while increasing the activities of superoxide dismutase (SOD) and catalase (CAT) represented by 64.87% and 22.93%, respectively. RT-qPCR analysis revealed that FBBE-PS could up-regulate the lipid metabolism-related genes such as *ppar-α*, *acox-1* and *cpt-1α*, and oxidation-related genes such as *nrf2*, *ho-1*, *nqo-1*, *sod1*, *cat*, etc. The metabolomics analysis indicated that FBBE-PS could alleviate lipid deposition by inhibiting the biosynthesis of unsaturated fatty acids, which is consistent with the downregulation of *scd-1* expression. It is demonstrated that fermentation can alter the properties and physiological activities of polysaccharides in barley bran, and FBBE-PS exhibited an alleviating effect on lipid deposition and oxidative stress in high-fat cells.

## 1. Introduction

High-fat diets lead to lipid accumulation and lipid metabolism disorders, which are considered to be an important cause of metabolic syndromes such as obesity, non-alcoholic fatty liver disease (NAFLD), hyperlipidemia, etc. [1]. Meanwhile, lipid accumulation usually induces the occurrence of oxidative stress, which leads to mitochondrial dysfunction and the increase of reactive oxygen species (ROS), having adverse effects on human health [2]. It is of great significance to explore safe and effective natural ingredients to improve the metabolic syndromes caused by high-fat diets, so as to prevent lipid accumulation and reduce the occurrence of chronic diseases through dietary intervention.

Barley bran, a by-product of barley processing, is rich in functional substances including dietary fiber, phenolics, etc. [3]. It has shown positive health benefits in reducing chronic diseases [4]. Fermentation is beneficial to improve the nutritional quality and functional characteristics of cereals [5]. Our previous studies also confirmed that the fermentation by *Lactiplantibacillus plantarum* dy-1 could promote the functional properties of whole barley flour significantly, thereby inhibiting lipid deposition and regulating lipid metabolism [6]. Thus, it is meaningful to further explore the functional composition of fermented barley bran and its lipid-lowering activity.

Polysaccharides possess healthy characteristics in the form of dietary fiber and prebiotics [7,8]. Bioactive polysaccharides from cereals, especially β-glucan and arabinoxylan have confirmed their physiological functions and exhibited beneficial effects on maintaining the blood glucose, insulin and cholesterol levels remarkably [9,10]. Furthermore, it was found that the lipid-lowering activity of fermented barley β-glucan was higher, indicating that fermentation is an effective way to improve its physiological activity [11]. This indicated that fermented barley bran and its polysaccharides might also possess lipid-lowering abilities. Therefore, in the present study, the effect of fermentation on polysaccharides of barley bran was analyzed, and HepG2 cells induced by sodium oleate were used to investigate the regulation of polysaccharides from barley bran extract fermented with *Lactiplantibacillus plantarum* dy-1 (FBBE-PS) on lipid deposition and oxidative stress. Then metabolomics were performed to reveal the regulatory mechanism of FBBE-PS on high-fat HepG2 cells.

## 2. Materials and Methods

### 2.1. Sample Preparation

Barley bran was provided by Jiangsu Ruimu Biotechnology Co., Ltd. (Yancheng, China); *Lactobacillus plantarum* dy-1 (CGMCC 6016) was isolated and identified by the laboratory. Analytical grade reagents such as sodium hydroxide, etc. were purchased from Sinopharm Chemical Reagent (Shanghai, China) Co., Ltd. Regents such as methanol, acetonitrile, etc. used for chromatographic analysis were purchased from ANPEL Laboratory Technologies Int. (Shanghai, China). Ammonium acetate was purchased from Merck (China) Ltd. (Shanghai, China), ammonia was purchased from ThermoFisher (China) Scientific (Shanghai, China). Reagents for cell culture such as DMEM (Dulbecco’s modified eagle medium) medium, fetal bovine serum (FBS), etc. were purchased from Thermo Fisher Scientific. Trypsin and Oil Red O were purchased from Sangon Biotech (Shanghai, China) Co., Ltd. Sodium oleate was purchased from Kunchuang Technology Development Co., Ltd., Xi’an, China.

The sieved barley bran was filtered through a 30-mesh sieve and was mixed with water at a ratio of 1:7, and then *L. plantarum* dy-1 (1 × 10^7^ CFU/mL) was added. The mixture was shaken and fermented for 24 h at 31 °C. Then the broth was centrifuged at 9500 r/min for 20 min at 4 °C and the supernatant was freeze-dried by an FD-8 vacuum freeze dryer (Beijing Bio-cool Lab Apparatus Co., Ltd., China) to obtain the fermented barley bran extract with *Lactiplantibacillus plantarum* dy-1 (FBBE) powder. The raw barley bran extract (RBBE) powder was prepared without the addition of *L. plantarum* dy-1.

The preparation of polysaccharides referred to the method in [12,13] with a few modifications. Firstly, the crude polysaccharides were extracted twice from RBBE and FBBE with water at 80 °C for 1 h. After cooling to room temperature, 70% ethanol was added to the solution and it was left overnight at 4 °C. The precipitate was obtained by centrifugation and dissolved in water and stirred at 80 °C for 40 min. Then the protein was removed by Sevage reagent (n-butanol: chloroform = 1:4) and three volumes of anhydrous ethanol were added for precipitation. The precipitate was resuspended in distilled water, centrifugated and freeze-dried to obtain the RBBE-PS (polysaccharides from raw barley bran extract) and FBBE-PS (polysaccharides from fermented barley bran extract with *Lactiplantibacillus plantarum* dy-1).

### 2.2. Analysis of Polysaccharides

#### 2.2.1. The Determination of the Molecular Weight of Polysaccharides

The content of total polysaccharides was detected according to the phenol-sulfuric acid method referred to by Shang et al. [13]. The determination of molecular weight was performed by a high-performance size exclusion chromatography (HPSEC, Agilent Technologies Inc., Santa Clara, CA, USA) connected with a refractive index detector (RI) and a multi-angle laser light scattering detector (MALLS), referring to the method of He et al. [14] The concentration of polysaccharides was prepared as 1 mg/mL, which was filtered through 0.22 μm filter for analysis. The separation column was CarboPac PA10 (250 mm × 4 mm, Thermo Fisher Scientific, Santa Clara, CA, USA) and the column temperature was 30 °C. The mobile phase was 0.1 M of NaCl solution, the flow rate was 0.5 mL/min and the injection volume was 25 μL.

#### 2.2.2. The Determination of Monosaccharide Composition of Polysaccharides

Five milligrams (±0.05 mg) each of RBBE-PS and FBBE-PS were weighed and added to 1 mL of 2 M trifluoroacetic acid individually, then they were mixed and reacted at 121 °C for 2 h. The solution was dried with nitrogen and the residue was dissolved in 1 mL of distilled water for analysis. Then, the monosaccharides were analyzed by a Dionex ICS 5000 high-performance ion chromatography (HPIC) system coupled with a pulsed amperometric detector (PAD) (Thermo Fisher Scientific, Santa Clara, CA, USA). The separation was performed by a CarboPac PA-20 anion-exchange column (3 mm × 150 mm; 10 μm, Thermo Fisher Scientific, Santa Clara, CA, USA). The mobile phase A was 0.1 M of NaOH solution, mobile phase B was 0.1 M of NaOH solution containing 0.2 M of NaAc solution, the flow rate was 0.5 mL/min and the injection volume was 5 μL. The gradient elution program was as follows: 0 min: phase A/B (95:5, *v*/*v*), 30 min: phase A/B (80:20, *v*/*v*), 30.1 min: phase A/B (60:40, *v*/*v*), 45 min: phase A/B (60:40, *v*/*v*), 45.1 min: phase A/B (95:5, *v*/*v*), 60 min: phase A/B (95:5, *v*/*v*).

### 2.3. Cell Culture and Cytotoxicity Assay

The HepG2 cell line was provided by the Suzhou Institute of Biochemistry and Cell Biology, Chinese Academy of Sciences, Suzhou, China. The cells were cultured in DMEM medium supplemented by 10 % fetal bovine serum (FBS) in a 5% CO_2_ incubator at 37 °C (Forma 310, ThermoFisher Scientific, Santa Clara, CA, USA). The medium was replaced with fresh medium every 2 to 3 days and the cells were used within 10 in-house passages. The cell activity was evaluated by CCK-8 kit (Nanjing Jiancheng Bioengineering Institute, Nanjing, China) method.

### 2.4. Evaluation of Cellular Lipid Accumulation and Oxidative Stress

HepG2 cells were seeded at a density of 1 × 10^5^ cells/well in 12-well microplates for 24 h. The high-fat cells were prepared in 10% medium containing 0.5 mM of sodium oleate as the model group. A range of concentrations of RBBE, FBBE and FBBE-PS were added into the high-fat cells separately as the treatment groups. The cells cultured without sodium oleate were prepared as the control group. Then the cellular lipid accumulation was assessed by Oil Red O staining and triglyceride (TG) content. At the end of culture, the cells were washed twice with PBS (phosphate buffer saline) and mixed in 60% isopropanol for 30 min and stained with 60% Oil Red O in distilled water for 60 min. The unbound dye was removed by washing with PBS and 500 μL of isopropanol (100%) was added to each well for 10 min. Then the cells were observed and photographed by an Eclipse Ti-S fluorescence inverted microscope (Nikon Instruments (Shanghai) Co., Ltd. (Shanghai, China)) directly. In addition, the determinations of triglyceride (TG) content, cellular reactive oxygen species (ROS) level, superoxide dismutase (SOD) activity and catalase (CAT) activity were performed according to the introductions of the kits (Nanjing Jiancheng Bioengineering Institute, Nanjing, China).

### 2.5. Real-Time Quantitative PCR (RT-qPCR) Analysis

After extraction using a Takara MiniBEST Universal RNA Extraction Kit, the RNA concentration was measured with a NanoDrop 2000 nucleic acid analyzer (ThermoFisher Scientific, Santa Clara, CA, USA). The reverse transcription was performed using a Takara PrimeScript RT Master Mix kit to generate cDNA, and then real-time PCR assay was performed according to the SYBR^®^ Premix Ex Taq^TM^ kit method. A measured 2 μL of cDNA was mixed with primers and sterile water in proportion, then a 20 μL quantitative PCR reaction system was established to detect the gene expression [15]. The PCR program was set up as required by the kit instructions. The data were processed using the ^ΔΔ^CT threshold cycle method, and the expression was calculated using β-actin gene as internal reference. Three replicates were performed and the primers are listed in Table S1.

### 2.6. Cell Metabolomics Analysis

Three groups of HepG2 cells were prepared for metabolomics analysis, the control group was normal cells without sodium oleate and polysaccharides and the model group was high-fat cells induced by sodium oleate, while the FBBE-PS group was high-fat cells treated with 50 μg/mL of FBBE-PS. Before metabolomics analysis, the cultured HepG2 cells were collected by trypsin digestion and centrifugation. They were washed twice with pre-cooled PBS, then the cells were transferred to a 1.5 mL tube. Then, the cells were added to an equal ratio of extraction solution (methanol:acetonitrile:water = 2:2:1 (*v*/*v*), containing isotope-labeled internal standard mixture: nicotinamide-D4, acetylcholine-D9 chloride and L-leucine-D3 for positive ionization mode analysis and hippuric acid-D5, L-leucine-D3 and L-glutamic acid-13C5,15N for negtive ionization mode analysis), grinded at 35 Hz for 4 min, subjected to ultrasound for 5 min (ice-water bath) and centrifuged.

The chromatography conditions referred to the method of Liu [16]. A Vanquish UPLC (Ultra Performance Liquid Chromatography, ThermoFisher Scientific, Santa Clara, CA, USA) system was used for chromatographic analysis and the separation was performed at 4 °C by an ACQUITY UPLC BEH Amide column (2.1 mm × 100 mm, 1.7 μm, Waters Corporation, Milford, MA, USA). The mobile phase A was an aqueous phase containing 25 mmol/L ammonium acetate and ammonia, while mobile phase B was acetonitrile. The injection volume was 2 μL. The mass spectrometry conditions referred to the method of Li et al. [17] An Orbitrap Exploris 120 mass spectrometer (ThermoFisher Scientific, Santa Clara, CA, USA) was used for identification and the parameters were as follows: Sheath gas flow rate: 50 Arb, Aux gas flow rate: 15 Arb, Capillary temperature: 320 °C, Full MS resolution: 60,000, MS/MS resolution: 15,000, Collision energy: 10 ev/30 ev/60 ev in Stepped Normalized Collisional Energy (NCE) mode, Spray Voltage: 3.8 kV, (positive) or −3.4 kV (negative).

### 2.7. Statistical Analysis

All data were expressed as mean ± standard deviation (SD) and the significance was analyzed by t-test and one-way analysis of variance (ANOVA) using GraphPad Prism 9.0 (San Diego, CA, USA). The raw data of metabolites collected by Orbitrap Exploris were converted to the mzXML format and processed with an in-house program, which was developed using R and based on XCMS, for peak detection, extraction, alignment and integration. Then an in-house MS2 database (BiotreeDB) was applied in metabolite annotation. The cutoff for annotation was set at 0.3. The omics analysis was performed by MetaboAnalyst 4.0 and the figures were re-plotted by GraphPad Prism 9.0.

## 3. Results

### 3.1. Effect of Fermentation on Content and Molecular Weight of Polysaccharides

**Figure 1:**
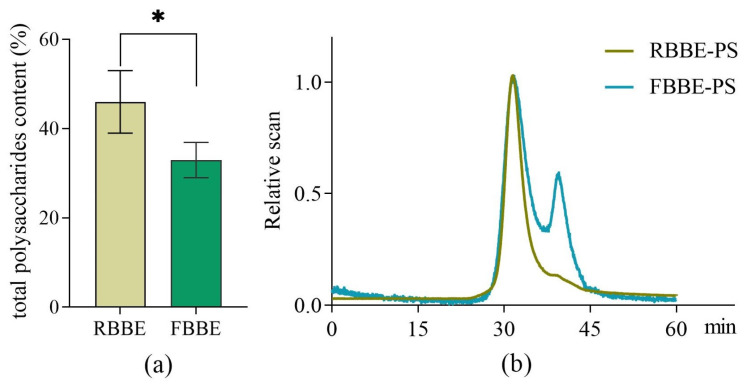
Effects of fermentation on the content of total polysaccharides in barley bran (**a**) and the HPSEC-RI-MALLS chromatograms (**b**) of polysaccharides from unfermented (RBBE-PS) and fermented barley bran extract (FBBE-PS). * *p* < 0.05.

As shown in Figure 1a, compared with RBBE, the total polysaccharides content of FBBE was reduced from 46.02% to 32.96%. This was because part of the fermentable sugars in the barley bran was utilized by *L. plantarum* dy-1. Meanwhile, molecular weight is an important factor affecting the bioactivity of polysaccharides. Studies have shown that the optimal activity of polysaccharide depends on its molecular weight [18]. Therefore, the weight-average molecular weight (Mw), number-average molecular weight (Mn) and molecular weight distribution (Mw/Mn) which related to the biological properties of polysaccharides [19] were analyzed by HPSEC-RI-MALLS. It was found that the chromatogram of RBBE-PS was a single symmetrical peak (Figure 1b), which indicated that the molecular weight distribution of RBBE-PS was uniform, while the molecular weight of FBBE-PS decreased from 3.396 × 10^5^ Da to 2.906 × 10^4^ Da and a second peak appeared in the chromatogram after fermentation (Table 1). This might be due to the enzymatic hydrolysis of lactic acid bacteria followed by the chain breaking of polysaccharides [20].

### 3.2. Effect of Fermentation on the Monosaccharide Composition of FBBE-PS

Monosaccharide composition is an important parameter affecting the biological activity of polysaccharides [19]. This is because monosaccharide composition can affect the senior structure of a polysaccharide, which has a significant relationship with the polysaccharide’s bioactivity [21]. In this study, the changes of monosaccharides affected by fermentation were analyzed by ion chromatography, and the results are shown in Table 2. It was found that both FBBE-PS and RBBE-PS were mainly composed of glucose, xylose, arabinose, galactose, etc., although their proportions were different. Compared with RBBE-PS, the molar ratio of glucose in FBBE-PS decreased by 54.53%, while the molar ratio of xylose and arabinose increased by 56.67% and 56.00%, respectively. This was due to the structural breakdown of barley bran cell walls caused by fermentation, the consumption of glucose and the release of xylose and arabinose [22]. In addition, mannuronic acid was detected in FBBE-PS instead of RBBE-PS, indicating mannuronic acid was the main metabolite in the fermentation process which was involved in the biological process of barley bran. It has been reported that uronic acid residues can alter the physicochemical properties and solubility of polysaccharides [23,24], which suggested that mannuronic acid may play an important role in the activity of FBBE-PS.

### 3.3. Effect of FBBE-PS on the Lipid Accumulation in HepG2 Cells

The cell cytotoxicity was measured to ensure the cell viability was not affected by sample addition. According to Figure S1, the cells were treated with RBBE and FBBE at a concentration less than 800 μg/mL, and FBBE-PS at a concentration less than 100 µg/mL.

**Figure 2:**
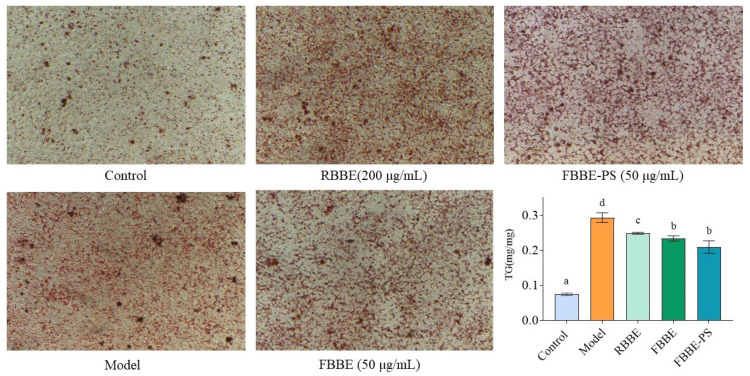
The micrographs (200×) of Oil Red O-stained cells and TG contents in HepG2 cells treated with 200 μg/mL of RBBE, 50 μg/mL of FBBE and 50 μg/mL of FBBE-PS. Data are presented as means ± SD, *n* = 5, different letters indicate significant differences between groups (*p* < 0.05).

Visual pictures of lipid accumulation were obtained by microscopic observation of Oil Red O-stained cells. As shown in Figure 2, compared with the control group, there were many more lipid droplets in the model group induced by sodium oleate, indicating that the high-fat cell model was successfully established. It could be clearly observed that the lipid droplets in the RBBE (200 μg/mL), FBBE (50 μg/mL) and FBBE-PS (50 μg/mL) groups were significantly less than those in the model group.

Meanwhile, as an important indicator for cellular lipid deposition, the content of triglyceride (TG) in RBBE (200 μg/mL), FBBE (50 μg/mL) and FBBE-PS (50 μg/mL) groups was detected and is shown in Figure 2. It was found that 200 μg/mL of RBBE, 50 μg/mL of FBBE and 50 μg/mL of FBBE-PS could reduce the TG content in high-fat cells significantly, although their TG levels were still much higher than normal cells in the control group. Combined with the results of Oil Red O staining, it could be preliminarily concluded that the lipid-lowering effect of FBBE was better than that of RBBE. Especially, 50 μg/mL of FBBE-PS group decreased the TG content by 21.62% compared with the model group, and there was no significance with the FBBE (50 μg/mL) group (*p* < 0.05), indicating that FBBE-PS might be the key active component to inhibit lipid deposition in high-fat HepG2 cells.

### 3.4. Effect of FBBE-PS on Fatty Acid Oxidation and Adipogenesis-related Genes in HepG2 Cells

The normal process of lipid metabolism is the balance between the synthesis of fatty acids and the beta-oxidation of fatty acids. Its related key enzymes included carnitine palmitoyltransferase-1 (CPT-1), aminocyclopropane carboxylic-1 (ACC-1), etc. In addition, *ppar-α*, as a transcription factor, can affect lipid metabolism by regulating the expression of genes involved in fat catabolism. During TG synthesis in hepatocytes, acetyl-CoA is converted to malonyl-CoA as a substrate, and the conversion is catalyzed by ACC1. Combined with fatty acid synthase (FASN), using acetyl-CoA and malonyl-CoA as starting materials, fatty acids are synthesized through a series of steps including acetyl condensation, reduction, dehydration and reduction reactions [25]. In the endoplasmic reticulum, saturated fatty acids are converted into monounsaturated fatty acids by stearoyl-CoA desaturase-1 (SCD-1) [26].

**Figure 3:**
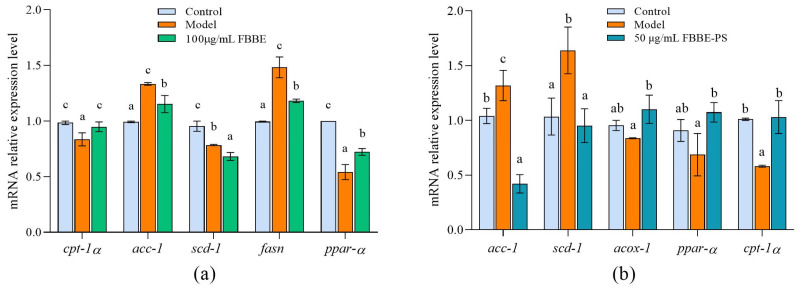
Effects of FBBE (100 μg/mL) (**a**) and FBBE-PS (50 μg/mL) (**b**) on the expression of genes related to fat metabolism in HepG2 cells. Data are presented as means ± SD, *n* = 3, different letters indicate significant differences between groups (*p* < 0.05).

Thus, the expressions of genes related to fatty acid oxidation and adipogenesis in cells were analyzed by RT-qPCR. As shown in Figure 3a, compared with the control group, the expression of *ppar-α* and *ppar-α*’s downstream target gene, *cpt-1a* in the model group, decreased to 54.13% and 84.84 % of the control group, respectively. Furthermore, 100 μg/mL of FBBE up-regulated the expression of *ppar-α* and *cpt-1a* significantly in high-fat HepG2 cells. For the adipogenesis-related genes, FBBE could decrease the expression of *acc-1*, *fasn* and *scd-1* compared with the model group. As shown in Figure 3b, compared with the model group the expressions of *ppar-α*, *acox-1* and *cpt-1α* in FBBE-PS (50 μg/mL) treatment cells up-regulated by 1.32 times, 1.56 times and 1.76 times, respectively. Additionally, the expressions of *acc-1* and *scd-1* in FBBE-PS group were significantly decreased to 31.88% and 58.08% of the model group, respectively. These results indicated that FBBE was able to promote fatty acid oxidation by activating *ppar-α* and inhibiting adipogenesis, thereby reducing fat deposition in high-fat cells. In addition, FBBE-PS could inhibit lipid accumulation in high-fat HepG2 cells by up-regulating genes involved in fatty acid β-oxidation and down-regulating genes related to adipogenesis.

### 3.5. Effect of FBBE-PS on Oxidative Stress in High-Fat HepG2 Cells Induced by Sodium Oleate

**Figure 4:**
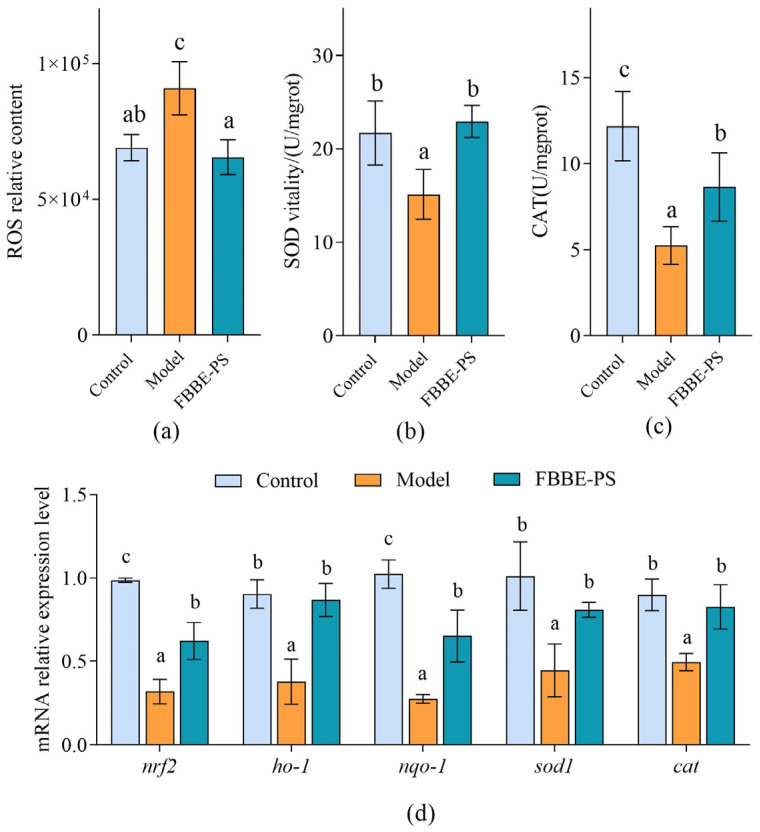
Effects of FBBE-PS (50 μg/mL) on intracellular ROS levels (**a**), SOD activity (**b**), CAT activity (**c**) and expressions of antioxidation-related genes (**d**) in HepG2 cells. Data are presented as means ± SD, *n* = 3, different letters indicate significant differences between groups (*p* < 0.05).

Excessive lipid deposition in cells can lead to mitochondrial beta-oxidation overload, resulting in intracellular oxidative stress disorder [27]. Therefore, the analysis of the effect of FBBE-PS on oxidative stress levels produced by high lipid cells is helpful to further confirm its effect on improving lipid deposition. Reactive oxygen species (ROS) are known to be critical for a variety of physiological processes at low levels, while they would impair cellular function, and damage cellular lipids, proteins and DNA at high concentrations [28]. As shown in Figure 4a, the intracellular ROS content in the high-fat HepG2 cells increased significantly compared with the control group, while 50 μg/mL of FBBE-PS could reduce the ROS level by 30.01% and there was no significant difference from the model group, exhibiting a good antioxidant activity.

As key antioxidant enzymes in cells, superoxide dismutase (SOD) and catalase (CAT) can prevent cell damage, block lipid oxidation in cells and protect cells from oxidative stress damage [29]; their activities are significant indicators to reflect the intracellular oxidative stress levels. As shown in Figure 4b, the activity of SOD in the model group was 15.13 U/mgrot, which was 69.72% of the control group, indicating that SOD activity was seriously affected by lipid accumulation. After 50 μg/mL of FBBE-PS treatment, the SOD activity increased to 22.93 U/mgrot, significantly, and returned to the level of normal cells in the control group. Meanwhile, compared with the model group, the CAT activity of FBBE-PS treatment cells was increased by 64.87%, which was closer to the normal cells (Figure 4c).

Based on the results obtained above, FBBE-PS has the ability to improve oxidative stress induced by lipid accumulation, which can enhance the activities of antioxidant enzymes and reduce the levels of ROS in high-fat cells. This might be one of the important reasons for its lipid-lowing activity. In addition, the expressions of oxidative stress-related genes also further confirmed the antioxidant effect of FBBE-PS. As shown in Figure 4d, compared with the model group, 50 μg/mL of FBBE-PS could upregulate the mRNA expressions of *nrf2*, *ho-1*, *nqo-1*, *sod-1* and *cat* remarkedly. In particular, the expressions of *ho-1*, *sod-1* and *cat* were not significantly different from those of normal cells in the control group. It was reported that fermented rice buckwheat (FRB) treatment could effectively ameliorate dyslipidemia, oxidative stress and chronic inflammation in high-fat diet-induced mice [30]. Additionally, dietary fermented wheat bran polysaccharides could upregulate transcription of antioxidant-related genes in the intestine of zebrafish [31], which confirms that the polysaccharides from fermented bran have an improvement effect on oxidative stress induced by high-fat diets.

### 3.6. Effect of FBBE-PS on Metabolism in High-Fat HepG2 Cells

**Figure 5:**
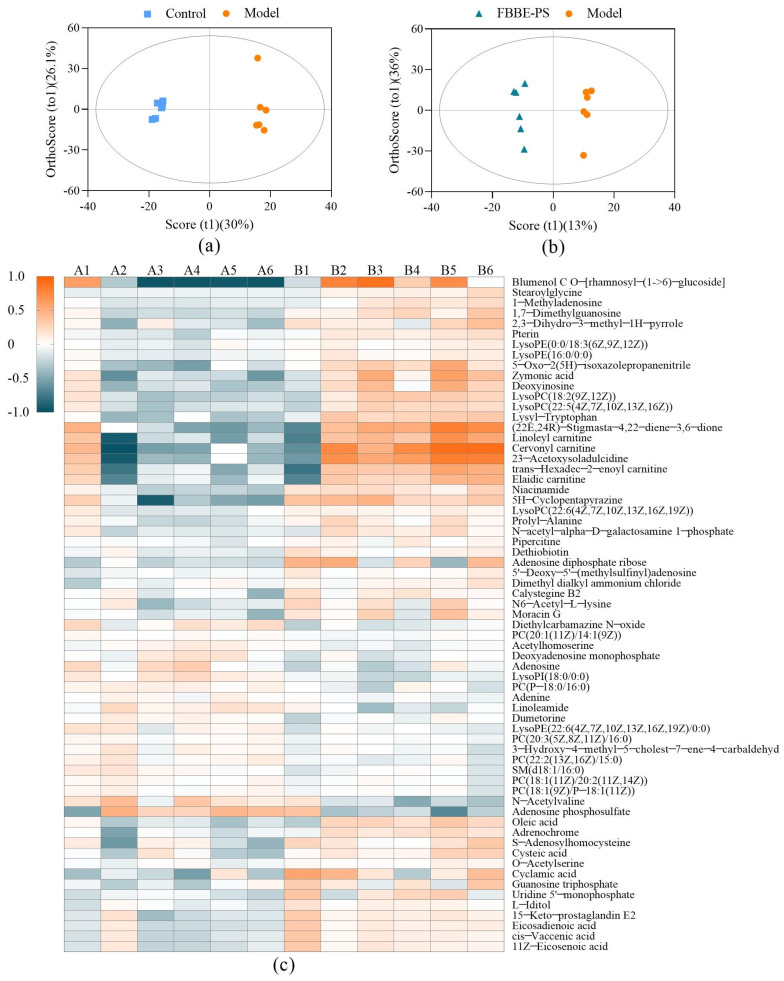
OPLS-DA plots of the control group vs. the model group (**a**) and FBBE-PS group vs. the model group (**b**). Heatmap plot (**c**) of differential metabolites in FBBE-PS group (A) and the model group (B).

The collected data of normal cells in the control group, high-fat cells in the model group and high-fat cells treated with 50 μg/mL of FBBE-PS were processed by MetaboAnalyst 4.0 for multivariate analysis. The OPLS-DA (orthogonal partial least squares-discriminant analysis) was performed and the differential metabolites of high-fat cells in FBBE-PS group and the model group were obtained in Table 3. The OPLS plots shown in Figure 5a,b indicate that the distinction between the three groups was obvious, and there were significant differences between the FBBE-PS group and the model group, indicating that FBBE-PS treatment altered the metabolic profile of HepG2 cells under the high-fat model.

The obtained metabolites identified in positive and negative ionization mode were analyzed by ANOVA, and those with VIP (variable importance in projection) > 1 and *p* value < 0.05 were defined as differential metabolites. As shown in Table 3, the differential metabolites were involved in amino acids such as lysyl-tryptophan, cysteine, etc.; organic acids such as pantothenic acid, taurine, cinnamic acid, etc.; and lipids such as deoxyadenosine monophosphate, cis-vaccenic acid, oleic acid, etc., which reflected the effects of FBBE-PS on the metabolic characteristics of high-fat HepG2 cells from the level of metabolites.

Then, a heatmap was plotted to show the differential metabolites (VIP > 1) in the FBBE-PS group and the model group. As shown in Figure 5c, the contents of differential metabolites between the two groups were quite different, especially since some of them were significantly reduced after FBBE-PS treatment, such as linoleoyl carnitine, cervonyl carnitine, etc. Similarly, the contents of these metabolites were also reduced in the control group. Acylcarnitine is generally synthesized by CPT-1, which is responsible for the transport of fatty acids into the mitochondrial matrix [32]. Incomplete fatty acid oxidation leads to increased acylcarnitine concentration [33], and the abnormal accumulation of acylcarnitine can accelerate the oxidation rate of fatty acids in mitochondria, resulting in oxidative damage [34]. It could be found that FBBE-PS might promote the decomposition of fatty acids and alleviate the lipid deposition by reducing the accumulation of acylcarnitine in high-fat cells.

Furthermore, LysoPC (20:4) and LysoPE (18:3) were hemolytic phospholipids, which are key components of cell membranes. It has been reported that lipid metabolism disorder could increase the level of hemolytic phospholipids [27]. As shown in Figure 5c, the content of LysoPC (20:4) and LysoPE (18:3) significantly decreased after FBBE-PS treatment, indicating that FBBE-PS could inhibit the metabolism of hemolytic phospholipids. Sphingomyelin (SM) has been reported to inhibit cholesterol absorption both in vivo and in cells, possibly caused by the slow absorption rate of cholesterol by cells due to the hydrolysis of SM [35]. Compared with the model group, the content of SM in FBBE-PS treatment group increased, indicating that it might reduce the absorption of cholesterol and alleviate the accumulation of lipids by promoting the biosynthesis of SM.

Much evidence has suggested that sulfur-containing amino acids have regulatory effects on lipid metabolism [36]. Compared with high-fat cells in the model group, cysteine content in the FBBE-PS-treated cells decreased, while S-adenosylhomocysteine content increased significantly, indicating that FBBE-PS might improve the metabolism of sulfur-containing amino acids in high-fat HepG2. In addition, oleic acid and cis-vaccenic acid were considered as the downstream products of de novo fat synthesis [37], hence their excessive contents in HepG2 cells could lead to the increase of TG. It is obvious that the contents of oleic acid and cis-vaccenic acid in FBBE-PS-treated cells were significantly lower than those in high-fat cells in the model group, indicating that FBBE-PS might ameliorate lipid accumulation by inhibiting fat synthesis.

**Figure 6:**
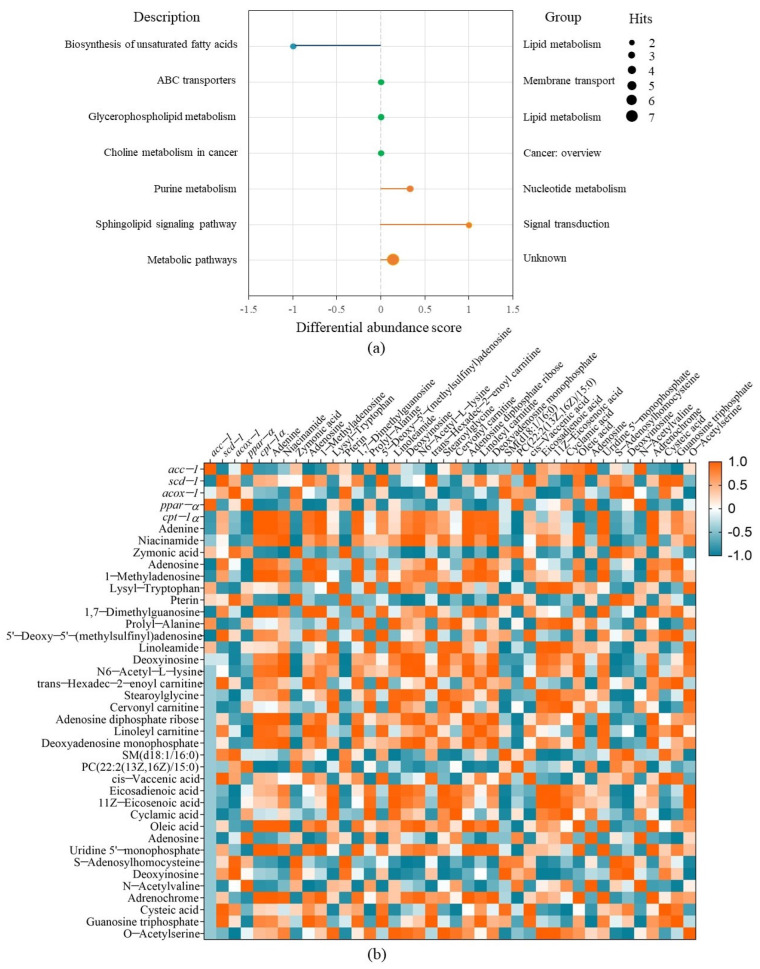
The differential abundance score diagram of FBBE-PS group vs. the model group for metabolic pathway analysis (**a**) and the correlation analysis between metabolites and lipid metabolism genes (**b**).

The metabolic pathway analysis for the differential metabolites were performed using KEGG (Kyoto Encyclopedia of Genes and Genomes). Then, a differential abundance score map reflecting the degree of metabolic pathway change was plotted, which can display the regulation of metabolic pathway and the metabolic type [38] (Figure 6a). It was shown that FBBE-PS mainly affected the unsaturated fatty acid anabolism pathway, sphingolipid signaling pathway, purine metabolism pathway, etc., in cells. Among them, the most significant effect was the down-regulation of unsaturated fatty acid anabolic pathway, in which oleic acid and eicosadienoic acid were the main differential metabolites changed by FBBE-PS treatment. Eicosadienoic acid was an ω6-fatty acid belonging to the family of free unsaturated fatty acids. Larger fat mass in high-fat cells led to the release of more eicosadienoic acid into metabolites. In high-fat HepG2 cells, the content of oleic acid and eicosadienoic acid was increased, and after FBBE-PS treatment, the content of them was significantly decreased. This was consistent with the result in Figure 5c. Additionally, palmitoleic acid and oleic acid synthesized by *scd-1* were the key substrates for the formation of complex lipids such as phospholipids, triglycerides and cholesterol esters, etc., and the decrease of *scd-1* expression in the present study was consistent with the trend of decreased oleic acid and TG content, indicating that FBBE-PS might improve lipid deposition by down-regulating the biosynthesis of unsaturated fatty acids.

In addition, compared with high-fat cells in the model group, the sphingolipid signaling pathway was up-regulated significantly. As reported, the sphingolipid signaling pathway is closely related to lipid metabolism. Excess lipid leads to increased accumulation of triacylglycerol, and the deposited triacylglycerol can be esterified to ceramide via the sphingolipid signaling pathway, leading to an increase in obesity [39]. Meanwhile, the purine metabolism pathway in FBBE-PS-treated cells was up-regulated, indicating FBBE-PS might interfere with lipid accumulation by affecting the metabolism of purines. Niemann et al. revealed that the purine inosine can be secreted by brown adipocyte apoptosis and stimulate energy expenditure in brown adipocytes by the cyclic adenosine monophosphate–protein kinase A signaling pathway [40]. As shown in Figure 5c, the content of adenine and adenosine increased in FBBE-PS-treated cells, while deoxyinosine content decreased. This might be one of the ways in which FBBE-PS exerts its lipid lowering activity.

Correlation analysis between the differential metabolites and gene expressions was carried out in combination with enrichment of metabolic pathways to further explain the mechanism of FBBE-PS treatment on improving cell lipid deposition. As shown in Figure 6b, *scd-1* and *acc-1* were positively correlated with the content of unsaturated fatty acids such as oleic acid and eicosadienoic acid. It confirmed that the inhibition of unsaturated fatty acid biosynthetic pathway is closely related to the alleviation of lipid deposition induced by high-fat diets. *scd-1* was a key enzymatic gene in the synthesis of monounsaturated fatty acids and *acc-1* was an upstream gene in the fatty acid synthesis pathway, regulating the synthesis of fatty acids [41]. Both oleic acid and eicosadienoic acid were members of the unsaturated fatty acid family, whose contents could be affected by *scd-1* (R^2^ = 0.854, 0.540, *p* < 0.01) and *acc-1* (R^2^ = 0.616, 0.586, *p* < 0.01) significantly. After FBBE-PS treatment, the expressions of *scd-1* and *acc-1* were significantly decreased compared with the model group. It could be concluded that FBBE-PS has the ability to regulate lipid deposition by inhibiting the synthesis of unsaturated fatty acids.

## 4. Conclusions

In this study, *Lactiplantibacillus plantarum* dy-1 fermentation could significantly affect barley bran polysaccharides content, molecular weight and monosaccharide composition, and the polysaccharides from fermented barley bran extract (FBBE-PS) could effectively inhibit lipid deposition of cells and improve cellular oxidative stress levels caused by high fat. The results indicated that fermentation could enhance the lipid-lowering ability of polysaccharides in barley bran by reducing its molecular weight and changing its monosaccharide composition. Through metabolomics analysis, it was revealed that FBBE-PS exerted lipid-lowering activity in high-fat HepG2 cells mainly by intervening in the biosynthesis of unsaturated fatty acid, sphingolipid signaling pathway, purine metabolism pathway, etc., in cells. This is helpful to further study the health promotion activity of fermented barley and other cereals. Furthermore, it is beneficial to the development and utilization of natural functional food raw materials by enhancing the activity of cereal polysaccharides through fermentation. In addition, there might be interactions between the active substances in *L. plantarum* fermented barley bran extract, such as polyphenols, polysaccharides, proteins or peptides, etc., which still require further investigation. It is necessary to screen, isolate and identify the active ingredients that inhibit lipid deposition in the future.

## Figures and Tables

**Table 1:** Molecular weight of polysaccharides from unfermented (RBBE-PS) and fermented barley bran extract (FBBE-PS).

Sample	Mw (g/mol)	Mn (g/mol)	Mw/Mn
RBBE-PS	3.396 × 10^5^	3.349 × 10^5^	1.014
FBBE-PS	2.906 × 10^4^	2.356 × 10^4^	1.233

**Table 2:** Monosaccharide compositions of polysaccharides from unfermented (RBBE-PS) and fermented barley bran extract (FBBE-PS).

NO	Component	Rt (min)	RBBE-PS (%)	FBBE-PS (%)
1	Fucose	3.375	0.24	0.37
2	Rhamnose	6.767	0.97	0.67
3	Arabinose	7.275	17.75	27.69
4	Galactose	9.217	10.54	15.91
5	Glucose	10.450	50.04	22.75
6	Xylose	12.242	14.46	22.64
7	Mannose	12.684	3.68	2.37
8	Mannuronic acid	14.600	—	6.46

**Table 3:** Differential metabolites of high-fat cells in FBBE-PS group and the model group.

NO.	Metabolites	Ionization Mode *	Molecular Formula	*p*-Value	VIP
1	Adenine	P	C_5_H_5_N_5_	0.0427	1.68
2	Niacinamide	P	C_6_H_6_N_2_O	0.0002	2.65
3	Zymonic acid	P	C_6_H_6_O_5_	0.0341	2.03
4	Adenosine	P	C_10_H_13_N_5_O_4_	0.0370	1.85
5	1-Methyladenosine	P	C_11_H_15_N_5_O_4_	0.0126	2.21
6	Lysyl-Tryptophan	P	C_17_H_24_N_4_O_3_	0.0007	2.38
7	Pterin	P	C_6_H_5_N_5_O	0.0007	2.46
8	1,7-Dimethylguanosine	P	C_12_H_17_N_5_O_5_	0.0024	2.61
9	Prolyl-Alanine	P	C_8_H_14_N_2_O_3_	0.0133	2.05
10	5’-Deoxy−5’- (methylsulfinyl)adenosine	P	C_11_H_15_N_5_O_4_S	0.0123	2.31
11	Linoleamide	P	C_18_H_33_NO	0.0102	1.81
12	Deoxyinosine	P	C_10_H_12_N_4_O_4_	0.0241	1.98
13	N6-Acetyl-L-lysine	P	C_8_H_16_N_2_O_3_	0.0421	1.73
14	Trans-Hexadec-2-enoyl carnitine	P	C_23_H_43_NO_4_	0.0280	1.27
15	Stearoylglycine	P	C_20_H_39_NO_3_	0.0202	2.31
16	Cervonyl carnitine	P	C_29_H_45_NO_4_	0.0145	2.04
17	Adenosine diphosphate ribose	P	C_15_H_23_N_5_O_14_P_2_	0.0460	1.96
18	Linoleyl carnitine	P	C_25_H_45_NO_4_	0.0258	1.50
19	Deoxyadenosine monophosphate	P	C_10_H_14_N_5_O_6_P	0.0493	1.69
20	SM(d18:1/16:0)	P	C_39_H_79_N_2_O_6_P	0.0202	2.18
21	PC(22:2(13Z,16Z)/15:0)	P	C_45_H_86_NO_8_P	0.0092	2.26
22	Cis-Vaccenic acid	N	C_18_H_34_O_2_	0.0138	2.23
23	Eicosadienoic acid	N	C_20_H_36_O_2_	0.0093	2.41
24	11Z-Eicosenoic acid	N	C_20_H_38_O_2_	0.0138	2.22
25	Cyclamic acid	N	C_6_H_13_NO_3_S	0.0328	2.21
26	Oleic acid	N	C_18_H_34_O_2_	0.0057	1.91
27	Adenosine	N	C_10_H_13_N_5_O_4_	0.0203	1.90
28	Uridine 5’-Monophosphate	N	C_9_H_13_N_2_O_9_P	0.0461	1.89
29	S-Adenosylhomocysteine	N	C_14_H_20_N_6_O_5_S	0.0461	2.20
30	Deoxyinosine	N	C_10_H_12_N_4_O_4_	0.0190	2.44
31	N-Acetylvaline	N	C_7_H_13_NO_3_	0.0116	2.10
32	Adrenochrome	N	C_9_H_9_NO_3_	0.0026	2.05
33	Cysteic acid	N	C_3_H_7_NO_5_S	0.0217	2.36
34	Guanosine triphosphate	N	C_10_H_16_N_5_O_14_P_3_	0.0397	2.05
35	O-Acetylserine	N	C_5_H_9_NO_4_	0.0218	2.20

* P: positive ion mode, N: negative ion mode.

## Data Availability

The data presented in this study are available in the main article and the supplementary materials.

## Supplementary Materials

The following supporting information can be downloaded at https://www.mdpi.com/article/10.3390/metabo13020223/s1, Table S1: Primers for real-time quantitative PCR; Figure S1: Effects of RBBE (**a**), FBBE (**b**) and FBBE-PS (**c**) concentration on HepG2 cell viability.
