# Supplementary material for: Metabolomics Reveal the Regulatory Effect of Polysaccharides from Fermented Barley Bran Extract on Lipid Accumulation in HepG2 Cells
Source: Metabolites. 2023 Feb 3;13(2):223. doi: 10.3390/metabo13020223 (PMC9962758; doi:10.3390/metabo13020223)
Supplement: Supplementary file 1 [file metabolites-13-00223-s001.zip › metabolites-2178345-supplementary.pdf]

Supplementary Materials

Table S1. Primers for real-time quantitative PCR

Gene	Forward primer sequence (5′ to 3′)	Reverse primer sequence (5′ to 3′)
ppar-α	AGGCTGTAAGGGCTTCTTTC	GCATTTGTTCCGGTTCTTCTTC
acc-1	GCACCTGCTACTATTGCTACTC	CAGTCCCAGCACTCACATAAC
scd-1	TTGCCAGCTCTAGCCTTTAAATTCGTACCGCTGG CACATCAACTT	TCCTGGTAGCATTATTCAGTAGTTTTGGAGACTTTCT CCGGTCAT
acox-1	CCGCTATGATGGGAATGTGTAT	GTGACTTCAGGTGCTTGTAAGA
cpt-1α	TCCTGGTGGGCTACAAATTAC	ACAGCAGATCCATGGCATAATA
fasn	CTAGGTTTGATGCCTCCTTCTT	ATGGCTTCATAGGTGACTTCC
nrf2	ATGGATTTGATTGACATCCTT	CATGTTTTTCTTTGTATCTGG
ho-1	GGAACTTTCAGAAGGGCCAG	GTCCTTGGTGTCATGGGTCA
nqo-1	CCATTCTGAAAGGCTGGTTTG	CTAGCTTTGATCTGGTTGTC
sod1	AGGTGTCTTTCGAAGATTCTGTGATC	TTTCTTCATTTCCACCTTTGCC
cat	GAGCAGCCCTGACAAAATGC	GGTAGGGACAGTTCACAGGTATCTG
β-actin	CCTTCCTGGGCATGGAGTCCTG	GGAGCAATGATCTTGATCTTC

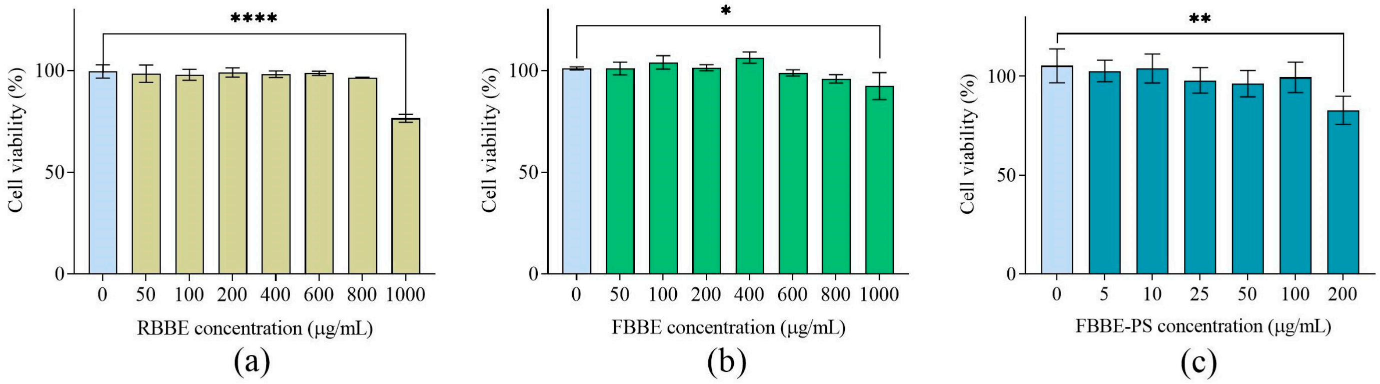

Figure S1. Effects of RBBE(a), FBBE (b) and FBBE-PS (c) concentration on HepG2 cell viability
